# Luminescence Sensing Method for Degradation Analysis of Bioactive Glass Fibers

**DOI:** 10.3390/s21062054

**Published:** 2021-03-15

**Authors:** Agata Baranowska, Marcin Kochanowicz, Aleksandra Wajda, Magdalena Leśniak, Jacek M. Żmojda, Piotr Miluski, Izabela Zgłobicka, Krzysztof J. Kurzydłowski, Dominik Dorosz

**Affiliations:** 1 Faculty of Mechanical Engineering, Bialystok University of Technology, 45C Wiejska Street, 15-351 Bialystok, Poland; a.baranowska@pb.edu.pl (A.B.); i.zglobicka@pb.edu.pl (I.Z.); k.kurzydlowski@pb.edu.pl (K.J.K.); 2 Faculty of Electrical Engineering, Bialystok University of Technology, 45D Wiejska Street, 15-351 Bialystok, Poland; m.kochanowicz@pb.edu.pl (M.K.); j.zmojda@pb.edu.pl (J.M.Ż.); p.miluski@pb.edu.pl (P.M.); 3 Faculty of Materials Science and Ceramics, AGH University of Science and Technology, 30 Mickiewicza, 30-059 Kraków, Poland; olawajda@agh.edu.pl (A.W.); mlesniak@agh.edu.pl (M.L.)

**Keywords:** bioactive glass fibers, luminescence, degradation, rare-earth, biomaterials

## Abstract

The effects of Sm^3+^ content on the optical properties and bioactivity of 13-93 bioactive glass were presented. Sm^3+^ doped glass fibers drawn from bioactive glass were analyzed in simulated body fluid (SBF) for the determination of ion release. Optical analysis of the Sm^3+^ ions in bioactive glass fibers was used for degradation monitoring. While the fibers were immersed in SBF solution, changes in their luminescence spectra under 405 nm laser excitation were measured continuously for 48 h. The morphology of the fibers after the immersion process was determined by SEM/EDS. It was shown that the proposed approach to the analysis of changes in Sm^3+^ ion luminescence is a sensitive method for the monitoring of degradation processes and the formation of hydroxycarbonate-apatite (HCA) layers on glass fiber surfaces. SEM/EDS measurements showed a significant deterioration on the surface of the fibers and the formation of HCA on 13-93_02Sm bioactive glass. The optical analysis of the time constant indicated that bioactive glass fibers doped with 2 %mol Sm^3+^ degrade at a rate almost five times slower than 13-93_02Sm.

## 1. Introduction

Larry Hench developed the first bioactive glass composition (45S5) in 1969 [1]. Its impressive biological properties have found wide application in the field of medicine, thus requiring the development of fibers with different glass forms. As the drawing of 45S5 fibers is problematic due to crystallization tendencies, intensive progress in the development of bioactive glasses led to the creation of new or upgraded bioactive glass compositions (such as 13-93) that offer improved thermal properties for the fiber-drawing process [2]. Nowadays, there is incessant demand for bioactive glass fibers with biomedical applications [3]. Due to their excellent mechanical and biological properties, they are sought as reinforcing elements in composites [4,5]. Moreover, from a clinical perspective, the fibers, due to their geometry and the possibility of controlled length and thickness, are an attractive material for the support of nerve regeneration [6]. 

Biomedical materials usually require monitoring of their condition over time, which should be possible in a non-invasive manner. Hence, lanthanide ion-doped materials have been widely proposed for various medical applications. In particular, the use of luminescent properties in sensors is popular and allows a wide range of possibilities [7,8,9,10]. Interesting optical properties of lanthanide ions offer opportunities for new applications and, due to their higher stability (in contrast to quantum dots), are already used in bio-imaging applications [11]. Despite the above, only a few reports have explored the use of rare-earth-doped bioactive glasses in the field of biomaterials. Most of them have reported on the application of rare-earth-containing glass materials as seeds for brachytherapy. The very first paper proposing the use of bioactive glasses containing samarium (Sm) ions was submitted by Roberto [12], and since then, other papers have proposed similar systems, but containing holmium, ytterbium, or yttrium. Other papers presented examples of hydroxyapatite [13], glasses, and nanofibers [14,15] doped with Eu^3+^ ions. In one paper [16], Gd^3+^- and Yb^3+^-doped bioactive glasses were presented as promising materials for medical applications. To the authors’ knowledge, the only paper that deals with lanthanide-doped bioactive fibers concerned nanofibers produced using an electrospinning method doped with Eu^3+^ and Tb^3+^ ions [17]. Among lanthanide ions, Sm^3+^ has luminescence properties that are interesting to analyze because of the relatively high quantum efficiency resulting from the wide energy gap (ΔE = 7000 cm^−1^) between the ^4^G_5/2_ emitting level and the underlying ^6^F_11/2_ level [18,19,20]. Additionally, its strong absorbance band at 405 nm allows for the excitation of glass materials with commercial lasers and the measurement of emissions in the visible spectrum (VIS) range. 

Summing up, currently, there is no single, universal technique for measuring the degradation of bioactive glass fibers. Existing methods cover measurements of weight loss, changes in pH, or the analysis of ions in solution. The use of optical methods to analyze the degree of fiber degradation can be a promising approach. Massera et al. [21] presented an optical method that relies on the measurement of changes in fiber light transmission as a function of immersion time. The proposed method for assessment of the degree of degradation with measurements of fiber transmission is a relative measurement, strongly dependent on the stability of the radiation source used, and may be subject to high error. 

This paper presents an investigation into the bioactivity and degradation properties of 13-93 glass and glass fibers doped with various concentrations of samarium (Sm) ions. The optical properties of Sm^3+^-doped bioactive glasses were examined. Bioactivity and microstructural measurements of doped glasses allow for the identification of lanthanide ions that affect formation of the HCA layer. Changes in the luminescent intensity of doped bioactive glass fibers enable control of the degradation process. The strongest emission band at the 600 nm wavelength has been observed under 405-nm laser excitation. The time constants for 0.2 and 2 %mol Sm^3+^ doped 13-93 glass fibers were determined and showed that a higher concentration of rare-earth ions affects bioactivity and slows down the degradation process. 

## 2. Materials and Methods

### 2.1. Materials and Glass Fiber Preparation

Samples of 13-93 bioactive glass -93 doped with Sm^3+^ ions were prepared from highly pure materials (Sigma-Aldrich, Saint Louis, MO, USA, 99.99%) using a standard melt-quenching method. All glass samples were doped with samarium ions at the expense of silicon dioxide. The original 13-93 molar composition is included in Table 1.

The homogenized set was put into a platinum crucible and melted at 1400 °C for 60 min. in an electric furnace (CZYLOK Company, Jastrzębie-Zdrój, Poland) under an air atmosphere. The molten glass was poured into a stainless-steel form, and a glass rod with 10-mm diameter was obtained. To reduce thermal stress, the material was annealed at 520 °C for 12 h in an electric furnace (CZYLOK Company). 

The obtained bioactive glass rod was fed into a tube furnace (SG Control, Newton, UK) with a suitably narrow temperature range. An SG Control 7.5 m height drawing tower was used to manufacture the glass fibers. Glass feed speed was controlled in the range of 0.2–1.6 mm/min. At this time, the glass rod melted and flowed out through the furnace outlet. Using a rotating drum, the outgoing stream of glass was drawn into fiber of a suitable diameter adjusted by the speed of the drum and the feed speed of the fiber preform. The drawing temperature was dependent on the glass composition; in this case, it was 1000–1100 °C.

### 2.2. Spectroscopic Studies in the Visible Range

Samarium-doped glasses were excited with a 405 nm laser (100 mW, Continous Wavelength (CW)). The luminescence spectrum was measured with a BROADCOM Qmini 2 Wide VIS spectrometer (San Jose, CA, USA) in the range of 525–750 nm with 1 nm resolution. The absorbance spectra in the range of 320–730 nm were determined using the aforementioned spectrometer. 

### 2.3. Bioactivity and Ion Release

Glass powders with a 50–100 µm particle size were soaked in simulated body fluid (SBF) at a ratio of 75 mg glass to 50 mL SBF. The SBF was prepared according to Kokubo’s method [22]. The samples were placed in polypropylene containers and then incubated at 37 °C for 4 h and 24 h. At the end of each period, the powders were separated by filtration and, in the case of materials immersed in SBF, washed with distilled water and acetone to terminate any ongoing reactions. The dissolution products were determined by measuring the ion concentrations with inductively coupled plasma optical emission spectrometry following the PN-EN ISO 11885:2009 standard (ICP-OES; Plasma 40, Perkin Elmer, Waltham, MA, USA).

### 2.4. Morphology 

To study morphology and surface reactions in vitro, the bioactive glass fibers doped with various concentrations of lanthanide ions were immersed in SBF prepared using Kokubo’s method [22]. Fiber samples were kept in closed containers and incubated in a thermal closet at 37 °C for 4, 8, 24, and 48 h. The volume of SBF used for testing was calculated with the following formula (Equation (1)):

V_s_ = S_a_/10, (1)

where V_s_ is the volume of SBF (ml), and S_a_ is the surface area of the fibers (mm^2^). In the experiment, 50 fibers of 4.5 cm in length were incubated in SBF. After the selected time (4, 8, 24, or 48 h), the fibers were taken out of the fluid and rinsed with water, then acetone, to stop any ongoing reactions.

Next, the morphologies of the dry fibers were observed with scanning electron microscopy. The glass fibers were mounted on a double-sided adhesive carbon tape on an aluminum-pin disc using tweezers. The observations were carried out with an ultra-high-resolution analytical dual-beam FIB-SEM tool (Scios 2 Dual Beam, Thermo Fisher Scientific, Waltham, MA, USA) equipped with an energy-dispersive full-range X-ray microanalysis system (NORAN System 7, NS7) using an acceleration voltage of 2 kV for the electron beams, without any additional conductive layer. During the observations, various magnifications from 500× to 50, 000× were used. Chemical analyses of the samples were obtained using a voltage of 30 kV, with an elemental range of 10 kV.

### 2.5. Experimental Procedure

**Figure 1:**
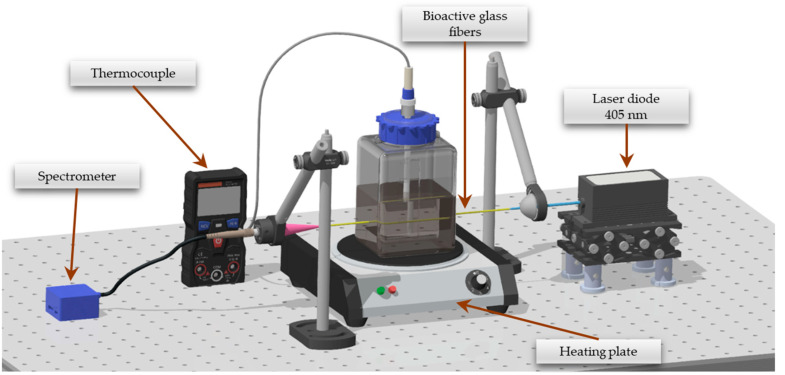
The measuring station for the optical method of determining the degradation process of bioactive glass fibers.

Fifty fibers, each of 13-93_02Sm and 13-93_2Sm glass with 130 μm ± 7 μm diameter, were immersed in SBF in a polypropylene container with their ends facing outwards. The interaction length was approx. 45 mm. Samarium-doped glass fibers were excited directly with a laser (100 mW, CW) generated at the 405 nm wavelength. The luminescence signal was collected at the end of the fibers using a BROADCOM Qmini 2 Wide VIS spectrometer (San Jose, CA, USA) in the 550–750 nm range with 1 nm resolution. Measurements were recorded every 1 min for 50 h. The container with SBF and fixed bioactive glass fibers doped with Sm^3+^ ions was placed on a IKA C-MAG HS 7 heating plate (IKA, Warsaw, Poland) with adjustable temperature and thermocouple. Automatic feedback facilitated regulation and stabilization of the liquid temperature throughout the procedure. The measuring station is illustrated below in Figure 1.

## 3. Results

### 3.1. Absorption Spectra

**Figure 2:**
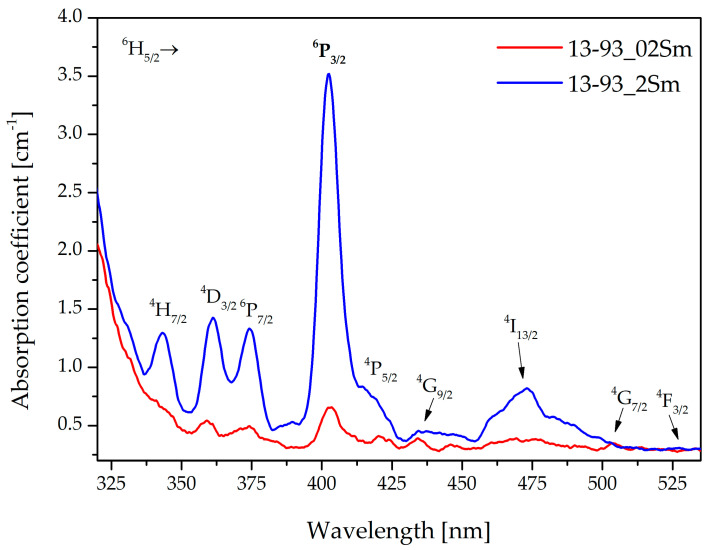
Absorption coefficient spectra of 13-93_02Sm and 13-93_2Sm.

Absorption spectra recorded for 13-93 bioactive glass doped with 0.2 and 2 %mol of Sm_2_O_3_ ions are shown in Figure 2. Doped glass absorption bands (especially for the 2 %mol sample) were observed at 344, 376, 404, 418, 437, 477, 501, and 528 nm, which correspond to the transitions from the ground ^6^H_5/2_ level to various excited ^3^H_7/2_, ^4^D_3/2_, ^6^D_7/2_, ^6^P_3/2_, ^4^P_5/2_, ^4^G_9/2_, ^4^I_13/2_, ^4^G_7/2_, and ^4^F_3/2_ states, respectively [19,23]. The band with the highest absorption coefficient value, which corresponds to the ^6^H_5/2_ → ^6^P_3/2_ transition, was applied for the excitation of the samarium-doped glasses. The use of radiation from the laser (λ_exc_ = 405 nm) contributed to the effective pumping of the studied glasses as well as the doped fibers. 

### 3.2. Luminescence Spectra

**Figure 3:**
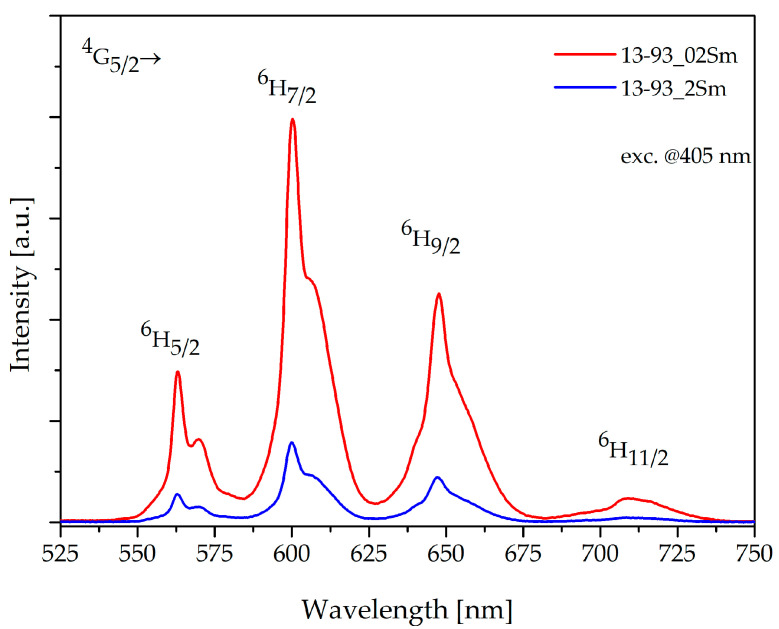
Luminescence spectra of 13-93 bioactive glass doped with 0.2 and 2 %mol Sm^3+^, λ_exc_ = 405 nm.

The luminescence spectra of 13-93 bioactive glass doped with 0.2 and 2 %mol Sm_2_O_3_ ions excited at 405 nm are shown in Figure 3. Fabricated glasses are characterized by intense reddish-orange luminescence that corresponds to the emission bands from the initial ^4^G_5/2_ to the lower states of ^6^H_J_, where J = 5/2, 7/2, 9/2, and 11/2 [20].

**Figure 4:**
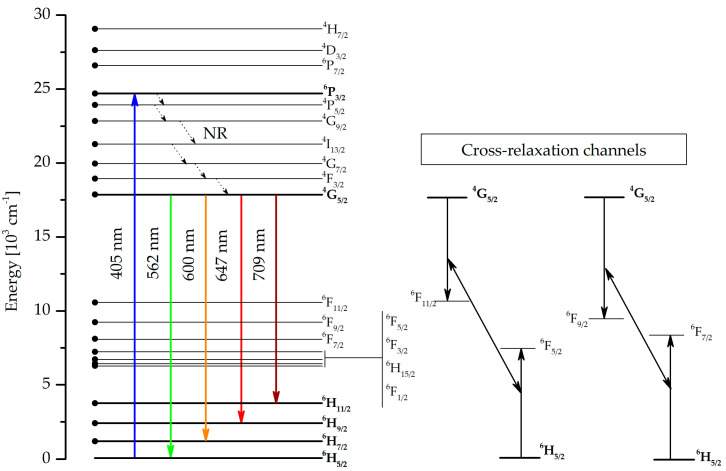
Energy level diagram of Sm^3+^ with emission and cross-relaxation channels [18,19].

The characteristic luminescence covers the emission bands at 562 nm, 600 nm, and 647 nm and also a relatively weak band at 709 nm. A significant decrease in intensity was recorded for the strongest emission band, which corresponds to ^4^G_5/2_ → ^6^H_7/2_ at 600 nm, is associated with the glass doped with 2 %mol Sm_2_O_3_, and can be attributed to the self-quenching effect. According to [18], the fluorescence intensity of the glasses increases up to 0.5 %wt. Sm^3+^ ion concentration, while further addition of samarium ions leads to luminescence quenching. Thus, as a result of doping bioactive glass material with 0.2 and 2 %mol of Sm^3+^ ions, it was determined that stronger luminescence intensity is obtained with a lower concentration of rare-earth ions. Concentration-dependent luminescence quenching is typically due to the interaction between ions; additionally, the closely spaced energy levels permit cross-relaxation channels (Figure 4) and non-radiative relaxation at higher concentrations [18]. However, it should be noted that this relatively high content of Sm_2_O_3_ was used intentionally in order to analyze the dopant effect on bioactive properties. Figure 4 presents the energy level diagram for Sm^3+^ ions with marked radiative transitions in the visible region and their possible cross-relaxation channels. Some of the presented energy levels (^6^F_11/2_, ^6^F_9/2_, ^6^F_7/2_, ^6^F_5/2_, ^6^F_3/2_, ^6^H_15/2_, and ^6^F_1/2_) correspond to absorption bands in the near-infrared region, which were not measured in the samples examined. Also cross-relaxation processes for the Sm^3+^ ions may take place as follows: (^4^G_5/2_; ^6^H_5/2_)→(^6^F_5/2_; ^6^F_11/2_) and (^4^G_5/2_; ^6^H_5/2_) →(^6^F_9/2_; ^6^F_7/2_) [18,20,24]. On the other hand, from Figure 4 it can be seen that emissions from the higher multiplets ^4^P_5/2_, ^4^G_9/2_, ^4^I_13/2_, ^4^G_7/2_, and ^4^F_3/2_ were not observed due to fast non-radiative transitions from the aforementioned ^4^G_5/2_ level [20].

**Figure 5:**
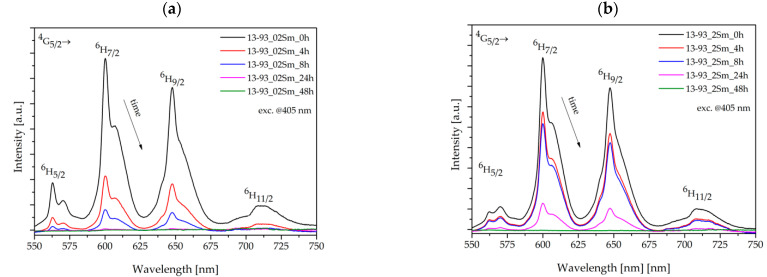
Emission spectra for (**a**) 13-93_02Sm and (**b**) 13-93_2Sm bioactive glass fibers immersed in simulated body fluid (SBF) for 0, 4, 8, 24, and 48 h.

In Figure 5 we can observe the luminescence spectra of the bioactive fibers doped with various concentrations of samarium ions before and during the degradation process, at specific time intervals: 4, 8, 24, and 48 h. It is seen that optimization of Sm^3+^ content allows for adjustments in sensor response as it is affected by the dissolution rates. The most intense transition, ^4^G_5/2_ → ^6^H_7/2_ (600 nm), was chosen for further experiments on bioactive glass fibers. 

### 3.3. Optical Measurements

**Figure 6:**
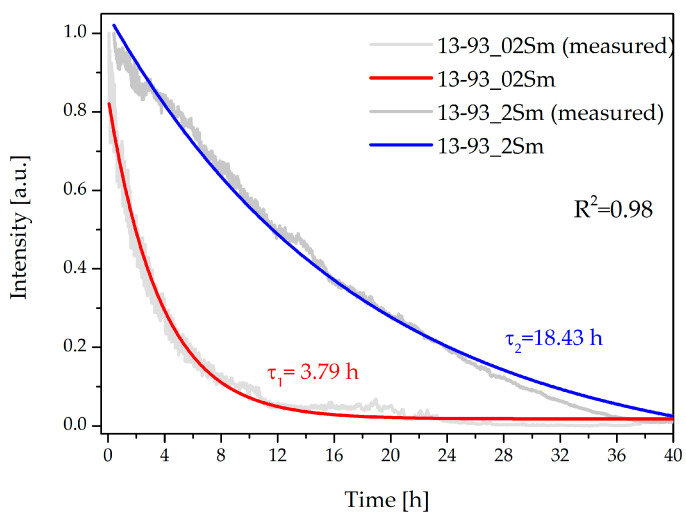
Characteristics of luminescence changes at 600 nm for 13-93 bioactive glass fibers doped with 0.2 and 2 %mol Sm_2_O_3_.

The luminescence changes observed at 600 nm for Sm^3+^-doped bioactive glass fibers excited at 405 nm, as a function of immersion time in SBF demonstrated the characteristics below (Figure 6). For 13-93_02Sm fibers, the time constant was much shorter (τ_1_ = 3.79 h) than for the 13-93_2Sm (τ_2_ = 18.43 h).

The coefficient of determination, R^2^, was 0.98, indicative of a good fitting for exponential decay as obtained with the following formula:

(2) $$I\left(t\right)=A_{1}\exp\left(-\frac{t}{\tau }\right)$$

where *τ* is the time constant and *A*_1_ is the fitting constant. Differences in the intensity changes between these two groups of fibers correlated with the concentrations of samarium ions. A higher volume of the dopant caused a slowdown in the degradation of the bioactive glass fiber. 

### 3.4. Morphology

**Figure 7:**
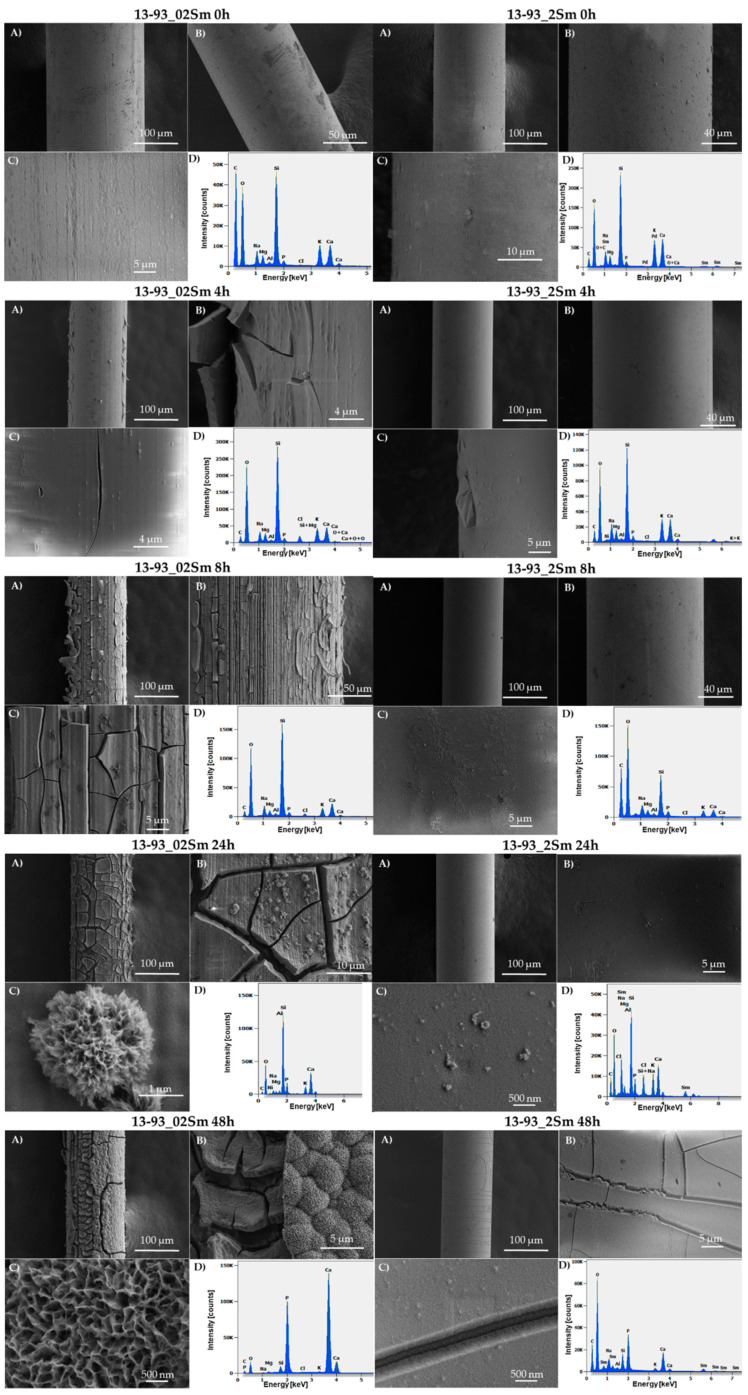
SEM micrographs of 13-93_02Sm and 13-93_2Sm bioactive glass fibers immersed in SBF for 0, 4, 8, 24, and 48 h, with EDS spectra of the surfaces. (**A**–**D**) are there to distinguish the scale in the images, and to clarify the description of a specific image.

Figure 7 shows micrographs obtained with scanning electron microscopy (SEM) at various resolutions and spectra obtained with energy-dispersive spectroscopy (EDS) from bioactive glass fibers doped with Sm^3+^ ions after 0, 4, 8, 24, and 48 h in SBF. The first two groups of micrographs represent bioactive 13-93_02Sm and 13-93_2Sm glass fibers before immersion in SBF. The fibers have a smooth surface without cracks or damage, which indicates that the correct temperature and speed were selected for the fiber drawing process. Energy-dispersive X-ray analysis provided an elemental analysis of the examined samples. In the EDS spectra of the initial samples (13-93_02Sm_0h and 13-93_2Sm_0h), Si is observed to be the dominant element, although sodium, magnesium, phosphorus, potassium, and calcium are also found, which corresponds to the composition of the glass (Table 1).

In the experiment with 0.2 %mol Sm_2_O_3_-doped fibers after 4 h in SBF, some surface cracks were observed. No noteworthy differences were found in the 13-93_2Sm glass fibers after the same period of incubation. The same condition characteristic of the initial material was observed even after 8 h. A small change in the form of slight cracks was detected a day after immersion of those fibers in SBF. In 13-93 glass fibers doped with 2 %mol of samarium ions, visible microscale cracks were detected after 48h of incubation in SBF. Moreover, significant changes were observed in the EDS spectra. The phosphorus content on the surface increased as a result of the degradation process in SBF. 

In the SEM micrographs, the formation process of HCA on the 13-93_02m glass fibers can be observed. As mentioned before, some microcracks on the surface can be observed just after 4 h of immersion in SBF, and after 8 h the entire fiber surface is cracked. In the next stage on the observed glass fiber area, cauliflower-like structures were noted. Then, after 48 h of incubation in SBF, the HCA layer completely covered the entire surface of the fiber. This was confirmed by EDS analysis showing the high content of phosphorus and calcium elements on the examined bioactive fiber surface. On the SEM micrographs (13-93_02Sm_48h-C, (Figure 7), it was possible to observe the structure of the bioactive HCA layer on the nanometer scale.

### 3.5. Bioactivity and Ion Release

In Table 2, the ion concentrations of SBF containing 13-93 doped with 0.2 and 2 %mol Sm_2_O_3_ under initial conditions, after 4 and 24 h. Observation of the changes in ion dissolution into the SBF from the bioactive glass allowed for the initial characterization of the behavior of the tested material in the environment of physiological fluid, i.e., in vitro measurements. The bioactivity test was performed using the indirect comparative method. Glass samples with 0.2 and 2 %mol Sm_2_O_3_ were analyzed. Changes in the glass during incubation in SBF were discernible in terms of the amounts of specific ions released. Based on that knowledge, the stages of hydroxy-carbonate apatite (HCA) layer formation on the glass surface could be predicted.

A significant increase was observed in released Si^4+^ ions, i.e., to 25 ppm after 24 h, which is associated with rapid cation exchange, leaching, and the formation of silanol groups [25]. For glass 13-93_02Sm after 4 h in SBF, the amount of silica in solution was approx. 13 ppm, whereas for the samples of glass doped with a higher concentration of samarium ions, the amount of silica was lower (approx. 8 ppm). Concentrations of released calcium, magnesium, and potassium ions were similar for each type of glass in the first 4 h, but after 24 h in SBF these ion concentrations were higher for the glass with 0.2 %mol of Sm^3+^. Conversely, decreases were observed over time in the concentration of phosphorus ions in SBF; this was indicative of precipitation and thus the formation of a CaP layer. Phosphorus was depleted faster in the 13-93_02Sm sample due to differences in the concentration of dopant in the bioactive glass. Interestingly, the results for the 13-93_2Sm sample revealed a heightened increase in the concentration of sodium ions in SBF.

## 4. Discussion

The sensing ability of obtained Rhenium (RE)-doped bioactive glasses and fibers is a novel aspect in biomedical engineering. Analyzing the Sm^3+^ energy diagram (Figure 4.) of doped bioactive glasses clearly confirms the existence of absorption and emission of light radiation in developed material. It was shown that a higher concentration of samarium ions does not guarantee a higher intensity of luminescence spectra in proposed bioactive glass. As observed with other silicate glasses, this is connected with the self-quenching phenomenon (Figure 3). The excitation of 13-93_02Sm and 13-93_2Sm bioactive glasses with the 405 nm laser demonstrated the higher luminescence intensity for glass doped with 0.2 %mol Sm_2_O_3_. This effect has to be considered particularly with respect to sensing applications, as the self-quenching mechanism results from a non-radiative process of energy transfer between Sm^3+^ ions through the cross-relaxation mechanism [26]. Moreover, the distribution of the energy states of Sm^3+^ ions (Figure 4) is favorable for cross-relaxation processes. The presence of these transitions leads to luminescence concentration quenching.

Taking the above into account, changes in luminescence intensity were used to predict microstructural changes in the bioactive glass fibers. After the 13-93_02Sm and 13-93_2Sm fibers were immersed in SBF solution, their microstructure changed, thus changing their optical properties as well. As a result of the chemical interactions—the release of ions, the formation of cracks on the surface and interiors of the glass fibers, and then the creation of an HCA layer [27], the degradation of the glass fiber as an optical medium for signal transmission increased [21]. The 13-93 glass fibers doped with both Sm^3+^ concentrations showed sufficient luminescence to be used to measure microstructural changes in contact with SBF. Depending on the concentration of dopant, the changes in luminescence signal varied. In the case of the glass fiber doped with a higher concentration of samarium ions (2 %mol), the time constant was longer. Luminescence signals changed less rapidly than in 13-93 with 0.2 %mol Sm_2_O_3_. The results can be explained not only by the different microstructural and textural changes in the materials with the surrounding SBF due to the composition of the bioactive glass fiber. RE-containing bioactive glasses dissolve more slowly than their parent glasses due to Si-O-RE bonds that are stronger than Si-O-Si bonds [16], and this effect can be more intense with increases in the concentration of rare-earth ions. In the examined bioactive fibers doped with 2 %mol Sm_2_O_3,_ the degradation process was slower than observed in 13-93_02Sm fibers due to the higher dopant content. On the other hand, the constant release of Sm^3+^ ions in the 2 %mol fibers causes a decrease in the RE concentration that may influence the luminescent emissions, diminishing the quenching effect. This has to be analyzed before adjusting for a proper RE concentration.

Degradation of the bioactive glass fibers starts with the release of the loosely bonded glass network (Ca^2+^ and Na^+^ cations) because of quick ion exchange with H^+^ from the physiological fluid. As a result, the pH level increases. This heightened pH level then promotes the delivery of silica from the network, and then the formation of Si-OH. Next, silanols collect and re-polymerize to form a silica-rich layer on the glass-fiber surface. In the next stage, the uninterrupted reaction of phosphate and calcium from the glass with the SBF creates hydroxyapatite (Ca_10_(PO_4_)_6_(OH)_2_), and soon after, crystallization processes form the hydroxycarbonate (carbonated hydroxyapatite—similar to the origin, hydroxyapatite (HPA)) on the glass-fiber surface [3,28]. The best illustrations for the mentioned reactions taking place in the examined active glass fiber are the SEM images with EDS spectra of the 13-93_02Sm and 13-93_2Sm (Figure 7), and in Table 2 showing the ions released from the glasses. Significant differences are noticeable depending on the dopant concentration in the glass and glass fiber. The incubation of the doped glass fibers in SBF after 4 and 8 h (for 13-93_2Sm, even after 24 h) showed very slight changes, in contrast to the optical measurements of luminescence decay, in which the first changes are noticeable already within the first hours. This proved that immediate changes in the bioactive glass fibers doped with lanthanide ions occurred in contact with SBF, and that there is a direct correlation between the microstructural and optical properties. Interestingly, after 48 h of incubation, with the 13-93_02Sm bioactive glass fibers in SBF, the whole surface was covered by the hydroxycarbonate apatite layer, which tended to fall off the surface, revealing the internal structure of the fiber.

The bioactivity test was prepared for 13-93 doped with 0.2 and 2 %mol Sm_2_O_3_ glasses to show ions releasing from the glass into the SBF. It can be seen that the examined glasses behaved comparably to ordinary bioactive glasses [25,29]. The large increase in the Si^4+^ concentration in the solution of the doped bioactive glass indicates that the glass powder was rapidly dissolved in the SBF. Thus, based on the SEM micrographs and the bioactivity test, it can be stated that bioactive glass fibers doped with lanthanide ions show bioactive properties, but depends on dopant concentration.

## 5. Conclusions

In this work, a novel approach to degradation process monitoring in bioactive glass fibers with a luminescent based method was proposed. The optical and biological properties of 13-93 glass fibers doped with various concentrations (0.2 and 2 %mol) of samarium ions were characterized. The results revealed that luminescent properties can be useful to monitor the degradation process of bioactive glass fiber with in vitro measurements. The Sm3+ ion emission was successfully used as an active sensor element. The presented data highlighted the fact that bioactive glass fibers doped with lanthanide ions exhibit sensing properties while preserving their biological activity.

This work has presented an optical method for measurement of degradation processes in bioactive glass and glass fibers. This approach allows in vitro observation of changes taking place in the material, in contrast to traditional methods. This study provides a foundation for the measurement of degradation processes not only in bioactive glass fibers, but also in composites, such as glass fibers in a polymer matrix. These findings hold promise for future applications in in vitro sensing devices for various biomaterials.

To describe changes in doped bioactive glass materials with more clarity and detail, further studies should aim to measure the extent of bioactivity over longer periods.

## Figures and Tables

**Table 1:** The molar composition of investigated glass fibers.

Glass Name	SiO_2_	P_2_O_5_	MgO	CaO	K_2_O	Na_2_O	Sm_2_O_3_
	[%mol]						
13-93	54.6	1.7	7.7	22.1	7.9	6	-
13-93_02Sm	54.4	1.7	7.7	22.1	7.9	6	0.2
13-93_2Sm	52.6	1.7	7.7	22.1	7.9	6	2

**Table 2:** Ion concentration of SBF containing 13-93 doped with 0.2 and 2 %mol Sm_2_O_3_ under initial conditions, after 4 and 24 h.

Time [h]	13-93_02Sm	13-93_2Sm
	**Si [ppm]**	
0	0.26 ± 0.01	0.26 ± 0.01
4	13.17 ± 3.97	8.34 ± 0.71
24	25.70 ± 0.77	25.29 ± 1.42
	**Ca [ppm]**	
0	87.70 ± 0.34	87.70 ± 0.34
4	96.49 ± 3.14	94.23 ± 2.23
24	111.53 ± 6.94	104.57 ± 8.39
	**P [ppm]**	
0	21.91 ± 0.24	21.91 ± 0.24
4	16.87 ± 1.01	16.56 ± 0.73
24	10.76 ± 0.35	12.32 ± 1.36
	**Na [ppm]**	
0	2398.25 ± 8.94	2398.25 ± 8.94
4	2376.53 ± 10.86	2412.56 ± 16.43
24	2461.70 ± 6.24	2537.08 ± 63.85
	**Mg [ppm]**	
0	28.95 ± 0.10	28.95 ± 0.10
4	32.24 ± 0.63	32.18 ± 0.34
24	37.61 ± 0.07	36.33 ± 0.97
	**K [ppm]**	
0	211.80 ± 1.75	211.80 ± 1.75
4	222.57 ± 3.20	222.43 ± 0.85
24	266.75 ± 1.14	250.70 ± 10.50

## Data Availability

Not applicable
